# An Information Manifold Perspective for Analyzing Test Data

**DOI:** 10.1177/01466216241310600

**Published:** 2024-12-20

**Authors:** James O. Ramsay, Juan Li, Joakim Wallmark, Marie Wiberg

**Affiliations:** 1 5620 McGill University, Montreal, Canada; 2 10055 Ottawa Hospital Research Institute, Ottawa, Canada; 3 8075 Umeå University, Sweden

**Keywords:** test information, surprisal, score index, expected sum score, scope, entropy, nominal model, spline functions, TestGardener

## Abstract

Modifications of current psychometric models for analyzing test data are proposed that produce an additive scale measure of information. This information measure is a one-dimensional space curve or curved surface manifold that is invariant across varying manifold indexing systems. The arc length along a curve manifold is used as it is an additive metric having a defined zero and a version of the bit as a unit. This property, referred to here as the scope of the test or an item, facilitates the evaluation of graphs and numerical summaries. The measurement power of the test is defined by the length of the manifold, and the performance or experiential level of a person by a position along the curve. In this study, we also use all information from the items including the information from the distractors. Test data from a large-scale college admissions test are used to illustrate the test information manifold perspective and to compare it with the well-known item response theory nominal model. It is illustrated that the use of information theory opens a vista of new ways of assessing item performance and inter-item dependency, as well as test takers’ knowledge.

## Introduction

A psychometrician communicates information from the administration of a test or scale to the stakeholders: test or scale designers, who need to assess the quality of test items; and test or scale takers and other assessors, whose primary concern is personal performance or experiential status. The data structure is a sequence of choices among two or more options for a sequence of items. The custom in the social sciences has been to lean heavily on probability theory in order to construct item and test taker graphical and numeric displays.

Probability has two serious information transmission liabilities. First, it is anything but linear except for a narrow window around 0.5 and may be either wasteful if the information is trivial or potentially disastrous if the message is extreme. Consequences of this include gambling casinos thriving and once in a hundred-year floods ignored. The second problem is that probabilities are intrinsically ratios and therefore cause many mathematical and computational problems when their denominators approach zero. Right answer counts or weighted scale sums converted to percentages are probabilities times 100.

In this article, we propose another approach, using three branches of mathematical analysis: information theory, manifolds, and functional data analysis. We propose to use a simple transformation to change probability into information in the technical sense. This enables direct comparisons among test items or between total test performances. This transformation also brings in computational benefit with information being defined over [0, +∞). The data analysis methods come from functional data analysis, which permits more flexibility in the estimation of item characteristics curves (ICCs), implying better fits to data than current parametric IRT models.

We propose to use full choice information, including if necessary the choice to omit an item or to return an illegitimate answer. In this way, the contamination of binary choice data by guessing can be avoided, and both minimum and maximum scores can be assigned even if a few answers to faulty questions are incorrect. The interactions between correct answers and their distracting options convey valuable insights into test taker performance and improve the accuracy of assessment.

Previous research within this area includes models permitting arbitrary flexibility in ICCs and option characteristic curves (OCCs) and fast estimation. Ramsay (1991) introduced kernel smoothing estimates of choice probability curves, and spline basis functions were used for this purpose along with ideas drawn from dynamic systems modelling in Rossi et al. (2002). Wiberg et al. (2019) compared parametric and spline-based models for binary-scored tests and showed that parametric models are too simple to adequately represent the data. Ramsay et al. (2020a) compared models for all options to their binary-scored counterparts and found that using distractor choices provided much more accurate expected sum scores. To use information about distractors can also been done with distractor analysis, see, for example, Haberman et al. (2019). Several IRT models for distractors have also been developed, see, for example, Briggs et al. (2006); Suh and Bolt (2010); Thissen and Steinberg (1984); Thissen et al. (1989); Wilson (1992). Several of these are extensions of or modifications of Bock’s (1972) nominal response model. Ramsay et al. (2020b) adapted the full-option spline model to rating scale data and demonstrated a large improvement in score accuracy over sum-scoring.

To be able to compare our proposed approach, we will use the well-known parametric IRT nominal model. The handbook edited by van der Linden (2016) details current IRT models for dichotomous, polytomous unordered nominal choices, or ordered rating scale data. Thissen and Cai (2016) provide detailed descriptions of models with the structure

(1) $$P_{im}\left(\theta_{j}\right)=\frac{\exp\left(a_{im}\theta_{j}+b_{im}\right)}{\sum_{l=1}^{M_{i}}\;\exp\left(a_{il}\theta_{j}+b_{il}\right)}$$

for item i,i=1,…,n option $m,m=1,\ldots ,M_{i}$ and $\theta_{j},$ j=1,…,N . This class, called the nominal model, includes most of the models for unordered multi-category data with unidimensional ability value θ that have appeared in the psychometric literature and therefore provides a convenient benchmark for evaluating the real data results in *Empirical Illustration: The Swedish SAT*.

The rest of the article is structured as follows. The next section describes how to transform probability to surprisal. The following section describes the concepts of information and the smooth low-dimensional manifold within a higher dimension space. *The Surprisal/Score Index Optimization Cycle* details an optimization cycle that alternates between manifold estimation given score indices and score index optimization given the manifold estimate. *Empirical Illustration: The Swedish SAT* illustrates various analyses using a part of a large-scale college admissions test assessing quantitative knowledge. Comparisons between the proposed information manifold perspective and the nominal model are also included in this section. The article ends with a discussion with some concluding remarks.

## From Probability to Surprisal

A non-negative real number *S*, that we call *surprisal*, can be constructed from a positive probability *P* as $S_{M}\left(P\right)=-\log_{M}\left(P\right)=\log_{M}\left(1/P\right)$ where *M* is an arbitrary log-base. Surprisal can be interpreted as quantifying the level of “surprise” of a particular event. An intuitive example is coin toss, where the probability of having head *n* times in a row is $P={\left(1/2\right)}^{n}$ , and the corresponding surprisal with base 2 is $S_{2}\left(P\right)=-\log_{2}\left(P\right)=\log_{2}\left(1/P\right)=n$ . The term surprisal for this transformation is natural since it quantifies the rarity of events in terms of real numbers. As events become more and more rare, their surprisal values grow in an additive way and thus offer a convenient way of subjectively gauging the consequences of rare but catastrophic events. While probability gets smaller and smaller to the point where it is difficult to discriminate, surprisal grows smoothly and additively. Conversely, large probability values near one, usually indicating an expected event, convert to unsurprising values close to or equal to zero.

**Figure 1.**
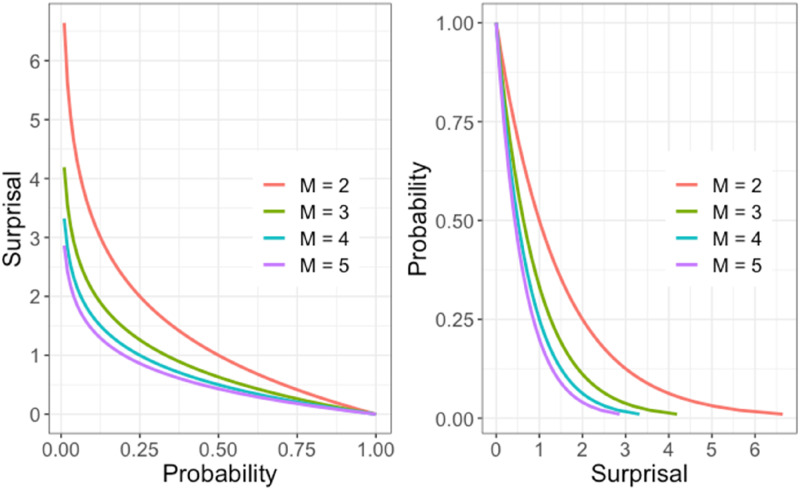
The relation between surprisal or information and probability for various values of base *M*.

Similarly, if an event is one among *M* possible outcomes rather than just two, then we change the log-base to *M* , so that events like a roulette ball with *M* pockets landing in the same pocket twice is simply two, and so on. A log-base 2 surprisal *S*_*2*_ and a base *M* surprisal *S*_*M*_ are related by $S_{M}=S_{2}/\;\log_{2}\;M$ . If *M* varies over test items, this transformation to a common binary scale may be useful. Figure 1 shows the relationship between surprisal and probability for a range of *M*-values.

Information theory is the mathematical representation of the transmission of messages through a communication system, and surprisal is a quantification of information. Three resources are Cover and Thomas (2006), Shannon (1948), and Stone (2022), and the last of these is especially suitable for a first contact with the topic. On an intuitive level, message transmission is what a multiple-choice test does; a choice of an option in an item is a message, and the communication system is the test. Within information theory, *S(P)* is called *self-information*, but we prefer the term *surprisal* introduced by Tribus (1961).

The surprisal transform is used extensively in statistics in the form of the log-odds transform, negative log likelihood, deviance, and in the theory of choice in mathematical psychology (Luce, 1959) where surprisal is referred to as the “strength” of a choice. It is the core of the maximum likelihood and Bayesian-based inference, which considerably preceded Shannon’s famous 1948 article by that of Fisher (1922). The classic text that argues for switching from probability to information theory is Kullback (1959).

### Information and Entropy

The information theory measure *entropy* summarizes the amount of information in a choice set by taking mean or expected value across choice surprisals.

(2) $$H_{i}=-\overset{M}{\underset{m}{\sum }}p_{im}\;\log\;p_{im}=\overset{M}{\underset{m}{\sum }}p_{im}s_{im}.$$

Entropy is maximized when $p_{im}=1/M_{i}$ and is consequently viewed as a measure of *disorder* or lack of structure. Entropy is minimized at zero and reflects certainty when every option probability except one is zero.

Since the amount of transmission of information from one surprisal vector to another is central to messaging, the *joint entropy* of two multinomial vectors of lengths $M_{i}$ and $M_{l}$ is assessed as the scalar

(3) $$J_{il}=\overset{M_{i}}{\underset{m}{\sum }}\overset{M_{l}}{\underset{n}{\sum }}p_{mn}^{il}s_{mn}^{il}$$

where $p_{mn}^{il}$ and $s_{mn}^{il}$ are the *joint* probability and surprisal values of two option choices *m* and *n* within items *i* and ℓ, respectively. The scalar symmetric summary of the *mutual entropy* or information intersection of two surprisal vectors, which is

(4) $$I_{il}=H_{l}+H_{l}-J_{il}.$$

Mutual entropy $I_{il}$ is small when the two multinomial variables vary independently but is equal to either *H*_*i*_ or *H*_*ℓ*_ when the two variables essentially measure the same choices. Mutual entropy is the information analogue of correlation and summarizes shared information across options as well as across items.

## From Flat Planes to Curved Manifolds

A manifold can be described as a smooth space that locally resembles a Euclidean space and is embedded within a higher dimension space. A space curve within a higher dimensional space is referred to as a *space manifold* and a two or higher dimensional surface as a *surface manifold*. Differentiable curves and surfaces within high dimensional spaces are curve or surface manifolds since they are essentially flat over tiny regions around a point. Both multinomial and information vectors of length *M* are manifolds of dimension M−1 within the vector space of *M*-vectors.

The following manifolds are essential in the proposed test analysis: surface manifolds defined by probability and surprisal vectors, and the curve manifold that either **P**(*θ*) or **S**(*θ*) defines when a multinomial or surprisal vector evolves smoothly over a single indexing variable *θ*.

### Three Psychometric Manifolds: $M_{P}$ , $M_{S}$ , and $M_{I}$

When searching for important structures in data, we usually assume that there is information in these structures that is large in some sense but low in dimensionality and smooth, and there is background noise information that is small, high dimensional, and complex. Two operations are required to use the manifold structure as a modelling object: charting and retracting. We first illustrate these two operations for PCA to introduce the related concept and then display the retractors for the probability and surprisal surface manifolds.

#### The Flat Principal Components Manifold $M_{PCA}$

The iconic manifold estimation method in data analysis is PCA, where a hyperplane of dimension *K* << *L* is defined by the eigenvalues and eigenvectors **D** and **V**, respectively, of a cross-product or covariance matrix of size *L*.

A position inside the hyperplane $M_{PCA}$ must be definable by a function *ϕ*, called the chart of the manifold, with values *ϕ*(**x**) ∈ $M_{PCA}$ where the indexing value **x** is of dimension *K*. The first *K* eigenvectors in an *L* by *K* matrix **V** serve this purpose because the linear transformation *ϕ*(**x**) = **Vx** of a vector **x** of length *K* will define a unique position **y** that is within the hyperplane. But any nonsingular rotation **VT**, where **T′T** = **I**, of the eigenvectors will also serve as indexing system, and the rotated vectors may well be more interpretable, so that $M_{PCA}$ has an infinity of alternative charts.

An essential part of a PCA is to identify points within the hyperplane corresponding to each data point **y** in the outer embedding space, an operation that we refer to as retraction. The projection operation **z** = **VV′y**, where **z** ∈ $M_{PCA}$ , is used for this purpose. PCA manifolds are flat, but for low-dimensional manifolds that are curved, bounded, and otherwise non-planar, the retraction operation can be more complex.

#### The Flat Probability Surface Manifold $M_{P}$

A set of probability vectors **P** of length *M* is a manifold, which we denote as $M_{P}$ , of dimension M−1 within the *M*-space of non-negative vectors. When *M* = 3, the set is flat equilateral triangle with vertices (1, 0, 0), (0, 1, 0), and (0, 0, 1), and higher dimensional vectors are equilateral hyper-planar simplices.

Psychometricians define a chart of the probability manifold $M_{P}$ in a way that emphasizes that a probability is inevitably a ratio. Let M by M−1 matrix Z satisfy the conditions $Z^{\prime }1_{M}=0_{M-1}$ and $Z^{\prime }Z=I_{M-1}$ , where $0_{M},1_{M}$ , and $I_{M}$ are column vectors of *M* zeros and ones and the identity matrix of order *M*, respectively. There are many ways to construct matrices **Z** with this orthonormal structure, including using the full QR-decomposition of $1_{M}$ and the Fourier and polynomial orthonormal series. Let vector **b** be an arbitrary vector of length M−1 , let vector **X** = −**Zb** and let vector **E** contain the values $e^{x_{m}}$ . Then **P** can be defined by $p_{m}=e_{m}/\left(\sum_{l}^{M}e_{l}\right)$ . This ratio charts a nonsingular multinomial manifold $M_{P}$ of dimension M−1 within the ambient space of non-negative vectors of length *M* using the chart index **b** of dimension M−1 . Division by the scalar value $\sum_{l}^{M}e_{l}$ retracts a positive vector **E** into the multinomial probability space $M_{P}$ . We assume non-singularity in our probability vectors, and this chart covers only the interior of the probability manifold because exponentials of finite vectors cannot reach zero or one.

#### The Curved Surprisal or Information Surface Manifold $M_{S}$

The charting function for surprisal is $S=X+1\left(\log_{M}\times \left(1^{\prime }\;M^{X}\;\right)\right)$ . Vector **X** is retracted into a surprisal vector by adding the scalar $\log_{M}\left(1^{\prime }M^{X}\right)$ to each of its elements.

**Figure 2.**
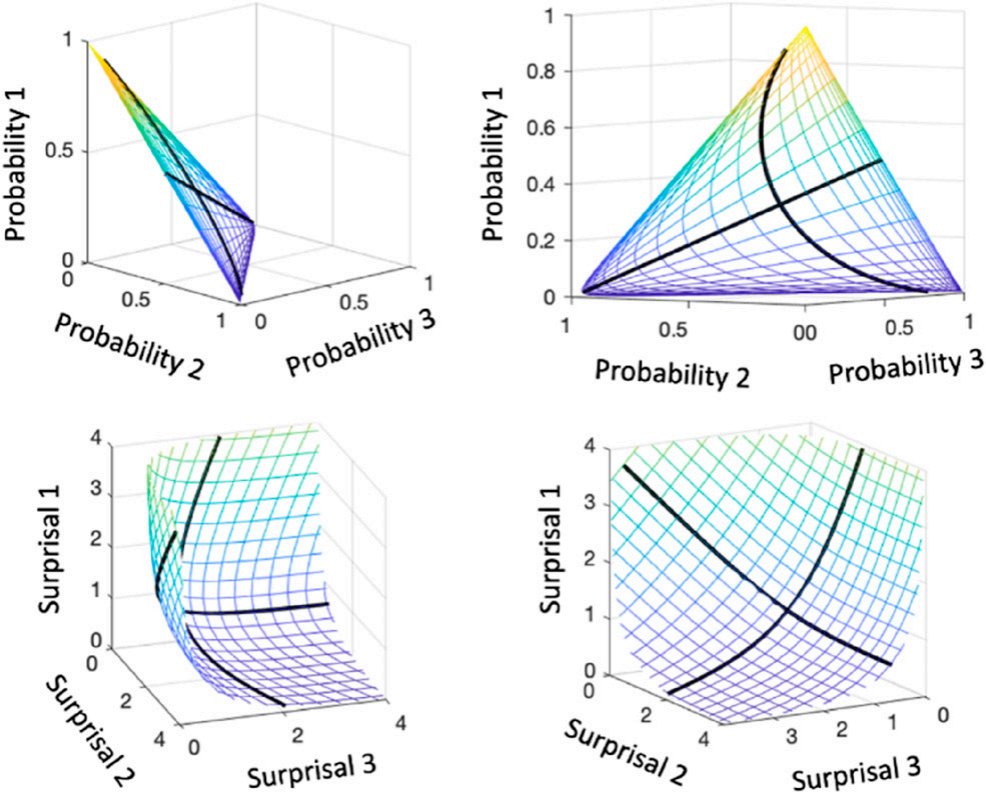
The top two panels display two views of the chart into the probability surface manifold of a two-dimensional mesh constructed from 21 points equally spaced over [-3,3]. The bottom two panels display two views of the chart of this mesh into the surprisal surface manifold. The thicker black curves are charts produced by fixing in turn a column of **b** to zero and varying the other. They are the analogues of coordinate lines on a plane.

Figure 2 compares the probability (top panels) and surprisal (bottom panels) manifolds in terms of a chart to each of a two-way mesh or grid defined by 21 points in [-3,3]. The probability grid lines are distributed over a planar region, but coalesce in the neighborhood of edges and vertices, so that the meaning of fixed differences is not constant. Moreover, we lose resolution near (0, 0, 0) from the perspective of our visual system at exactly where rare events are occur.

The surprisal chart sacrifices the planar shape in order to retain the Cartesian coordinate system within the surprisal manifold. The manifold is located in the positive orthant of the Cartesian space, and the point closest to the origin corresponds to probability 1/*M* or maximum entropy. Larger surprisal values are located more and more closely to the orthant boundary planes. A fixed difference means the same thing everywhere within the soup-bowl shaped surface manifold, and surprisal values are therefore on an additive scale.

#### The Space Curve Manifold $M_{I}$

**Figure 3.**
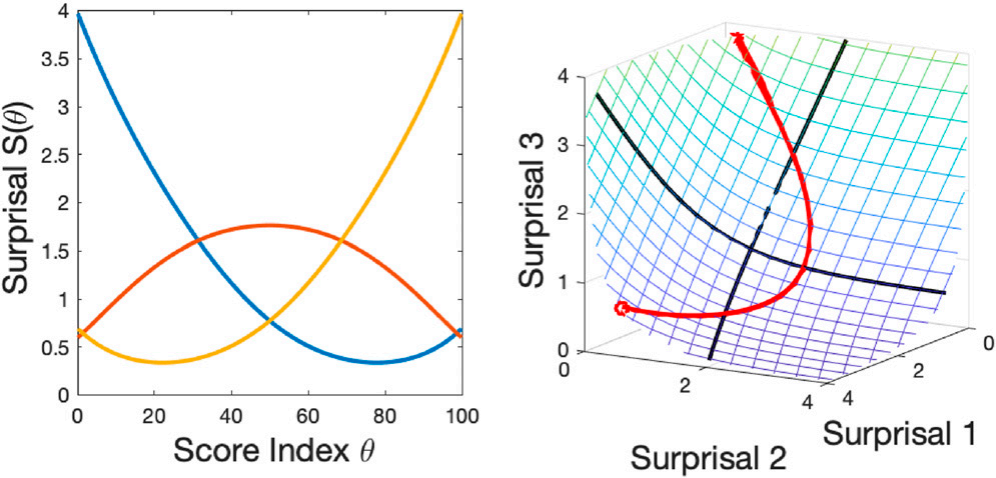
The left panel contains three random surprisal curves, not intended to reflect actual data. The right panel shows how the three curves vary jointly within the surprisal surface over a score index *θ*. The initial point is indicated by a circle.

We use surprisal for the more general situation where probability is a function of a quantity *θ*,

(5) $$S\left(\theta \right)=-\log_{M}\;P\left(\theta \right)\;\text{and}\;P\left(\theta \right)=M^{-S\left(\theta \right)}$$

which is the relation and its inverse between nonsingular multinomial vector **P** of length *M* and its surprisal equivalent **S**. As score index *θ* varies, **S**(*θ*) traces a one-dimensional curve or *surprisal space curve* within the vector space of dimension *M*. This curve manifold is the third that we consider. It plays a central role in our methodology, and we denote it by $M_{I}$ . Figure 3 illustrates a space curve generated by the three surprisal curves in the left panel and traced out within the surprisal manifold in the right panel.

Let b(θ) be an arbitrary real twice-differentiable vector function of θ of length M−1 and let X(θ)=−Zb(θ) . Vector **b**(*θ*) is a vector within a space of dimension M−1 and therefore is a chart of surprisal space for each value of score index *θ*. Pre-multiplication by matrix **Z** translates any **b**(*θ*) in a vector **X**(*θ*) that sums to zero for any *θ* and therefore retains the dimension of **b**(*θ*). The optimization cycle adjusts the elements of **b**(*θ*) using standard optimization software to chart the optimal value **X**(*θ*) for defining the best surprisal values for representing the choice data.

(6) $$S\left(\theta \right)=X\left(\theta \right)+1\left(\log_{M}\left(1^{\prime }M^{X\left(\theta \right)}\right)\right).$$

Then S(θ) can be defined in matrix notation as

Division by the scalar value $1_{M}^{\prime }M^{X\left(\theta \right)}$ , where X(θ) is an unconstrained real M -vector, is a *retraction* operation which pulls the $M^{X}$ 's into the M−1 dimensional manifold of probability vectors and the corresponding retraction for surprisal vectors is to add scalar $\log_{M}\left(1^{\prime }M^{X\left(\theta \right)}\right)1$ .

We used spline basis functions $B_{k}\left(\theta \right),k=1,\ldots ,K$ to represent coordinate functions as $b_{i}\left(\theta \right)=C_{i}\phi \left(\theta \right)$ where the coordinate matrix $C_{i}$ is $M_{i}-1$ by K and ϕ(θ) contains in column vector form the K B-spline functions. After considerable experimentation, it seemed that seven basis functions provided sufficient flexibility to model data structures but few enough to avoid over-fitting.

It seems natural to assume that there are test takers who lack all knowledge and thus are essentially below the test and others whose knowledge level is so far above the material being tested that they can be considered above the test. Spline basis functions are defined a closed interval, and the basis functions used in our analysis were defined over the interval [0, 100], chosen because the interval is already familiar to test takers as a percentage.

But we must also assume that no test has perfect test items, due to vague descriptions, descriptions that certain readers can interpret in unusual ways, questions about material that may not be taught and questions whose interpretation can be impacted by being by the test language not being the test taker’s first. A test analysis should have at least some capacity to detect problematic items and still assign 0 to the weakest and 100 to the best. The conventional sum scores fail completely for the SweSAT data, which is the data used in the later empirical illustration. It is implausible that only two among more than 50,000 examinees should get perfect scores for a mid-secondary level test and assign all scores some level of success due to guessing, cheating and other factors. The TestGardener analysis does have this capacity, but the nominal model does not because it in principle uses the whole real line.

We used the R package mirt (Chalmers, 2012) to estimate nominal model probability option curves instead of estimating surprisal data directly and computed surprisal curves for the model by subjecting each curve value to the surprisal transform. In this way, all the displays and operations available to our analysis are also available for the nominal model for direct comparisons. Note that the R package TestGardener (Ramsay & Li, 2021) can also estimate a version of the nominal model defined over the closed interval [0,100].

#### Scope: Arc Lengths $d_{i}\left(\theta \right)$ and $d_{I}\left(\theta \right)$ in the Curve Manifold $M_{I}$

Whatever the indexing set, positions along the space curve defined by a single item, usually called *the item information curve* $d_{i}\left(\theta \right)$ , can be computed by arc length by integrating the length or norm of the slope vector: where the variable of integration *u* is an index value. Because this integral depends only on the surprisal values of each of the *M* curves, the arc length *θ* that it computes is invariant over smooth one-to-one transformations of the index. The surprisal or information curve manifold for the test, called the *test information curve* $d_{I}\left(\theta \right)$ , is also defined for the entire multiple-choice test or rating scale having *n* items by the indefinite integral.

(7) $$d_{i}\left(\theta \right)=\int_{0}^{\theta }\sqrt{\overset{n}{\underset{i}{\sum }}\overset{M_{i}}{\underset{m}{\sum }}{\left[\frac{ds_{im}}{du}\right]}^{2}}du.$$

Since arclengths also retain the additive scale property of surprisal or information, they provide an easily interpretable measure of the amount of information accumulated at point *θ* measured in *M*-bits. Moreover, the total length of an *item information curve* or the total *test information curve* provides a measure of performance for the item or test, respectively, and the critical measure of information in an item or in a test is the norm of the slope vector, $ds_{im}/d\theta .$ Arc length along either an item or a test information curve to score index value *θ* measures the amount of information that test taker possesses that is covered by an item or the total test, respectively. We will use *information scope* for an amount of information covered by either an item or a test, in *Empirical Illustration: The Swedish SAT*.

The Fisher information analogue of (7) where θ is uniformly distributed is

(8) $$d_{F}\left(\theta \right)=\int_{0}^{\theta }\overset{n}{\underset{i}{\sum }}\overset{M_{i}}{\underset{m}{\sum }}{\left[\frac{ds_{im}}{du}\right]}^{2}du$$

and therefore only trivially different from surprisal.

## The Surprisal/Score Index Optimization Cycle

**Figure 4.**
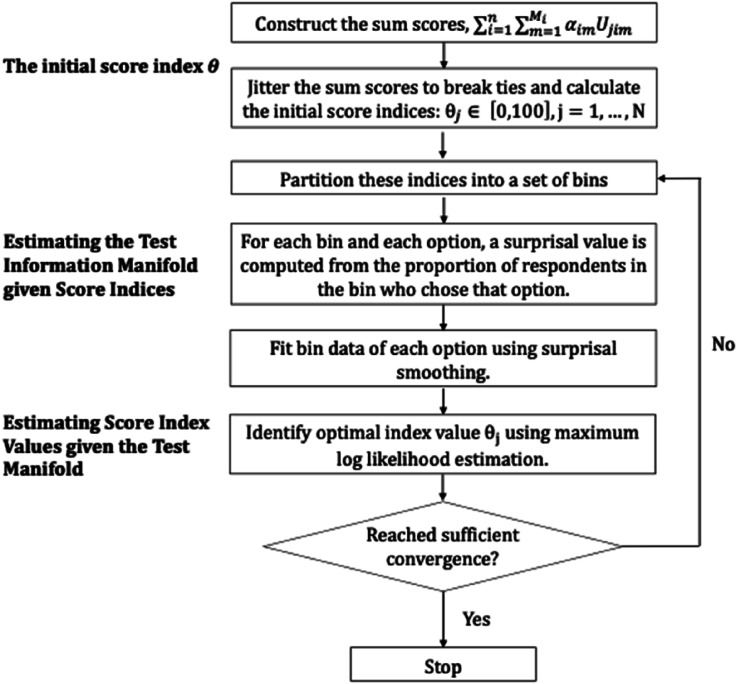
The initial two steps and the four steps within an analysis cycle.

Given the potentially large number of coefficient matrices **C**_*i*_ defining the *n* vectors **b**_*i*_(*θ*) and the even larger number of score indices ${\hat{\theta }}_{j}$ , combined with test scoring time constraints, parameter estimation must be both acceptably optimal and accomplished within a useful time frame. Figure 4 summarizes the initialization of cycles and the steps within each cycle.

As seen in the flow chart in Figure 4, we initialize the optimization by computing sum scores converted to percentage ranks. Next the sum scores are jittered, which means that the large number of tied sum scores inevitable in the use of integer-valued scores is removed by adding random values less than 0.5 to the scores to break up any possible dependencies in the data prior to ranking. An alternative strategy is to estimate person parameters by a low-dimension model such as the nominal and use these for percent ranking instead. Then the test information manifold given score indices are estimated, followed by the estimation of score index values given the test manifold. For computational details, please refer to the code on the github provided in the discussion section.

### Estimating Score Index Values $\theta_{j}$

The negative log likelihood objective function *F* for estimating a test taker’s position in $M_{I}$ is

(9) $$F\left(\theta_{j}\right)=\overset{n}{\underset{i}{\sum }}\overset{M_{i}}{\underset{m}{\sum }}U_{jim}S_{im}\left(\theta_{j}\right)=\overset{n}{\underset{i}{\sum }}S_{ij}^{c}\left(\theta_{j}\right)$$

where $S_{ij}^{c}$ is the surprisal for the chosen option for item *i* and test taker *j*. This equation is bilinear and therefore simpler than its probability counterpart, which brings substantial computational benefits. It also indicates that identifying the optimal value of *θ* resembles canonical correlation analysis because elements $s_{im}$ and *θ* are both being optimized. Unfortunately, it also implies that the value of $F\left(\theta_{j}\right)$ is of limited value in assessing the total model’s fit to the data, as is the case for canonical correlations. The size of F(θ) does not change much as we move from very simple models such as the nominal model to higher dimensional spline models using larger numbers of basis functions. The length of the total test information curve is preferable as a guide to choosing the number *K* of basis functions.

The gradient at the optimal value $\theta_{j}$ is

(10) $$\frac{dF}{d\theta_{j}}=\overset{n}{\underset{i}{\sum }}\overset{M_{i}}{\underset{m}{\sum }}U_{jim}\frac{ds_{im}}{d\theta_{j}}=\overset{M_{i}}{\underset{m}{\sum }}\frac{dS_{ij}^{C}}{d\theta_{j}}.$$

The bilinear structure of the log likelihood implies that, within the gradient, $dS_{ij}^{c}/d\theta >0$ pushes the current value of *θ* downwards and pushes it upwards for $dS_{ij}^{c}/d\theta <0$ . The optimal value is achieved with the sum of these slope values is 0. Surprisal derivatives of chosen option curves act as simple linear weights and can be presented to test takers as the point at which total negative slope is balanced by total positive slope. Test takers should find this scoring rationale of balancing success against failure as easy to understand as is the sum score.

It is important to note, however, that we observed that about 15% of the functions $F\left(\theta_{j}\right)$ exhibited multiple minima. A test scoring method must include a screen for such cases and bring these to the attention of the test scorer. Note, this limitation is also observed for classical IRT models. These multi-minima results seem natural since the test is assessing at least four recognizable skill clusters. A more thorough analysis would provide separate results for these four partitions.

### Estimating the Surface Manifold $M_{I}$

We estimated a smooth density function for the current score index values in order to construct bin boundaries that contain roughly equal frequencies. The number of bins *n*_*b*_ to use depends on the size of *N*. In the R package TestGardener, the default number of bins *n*_*b*_ are: *N* <500: *n*_*b*_ ≈ *N*/25, 500 ≤ *N* <10, 000: *n*_*b*_ ≈ *N*/50 and 10, 000 ≤ *N* : *n*_*b*_ ≈ 50. Within an item, bin proportions are computed for each option, and these proportions are then converted to surprisal values. A maximum surprisal value is used when a bin proportion is zero.

Next the *n*_*b*_ surprisal values for each of the $M_{i}$ choices are smoothed using a version of the least squares fitted smoothing splines that are adapted to the surprisal structure. We found that seven basis functions of order five without a smoothing penalty worked well. Note, the smallest possible number of spline basis functions is three, for which the order must also be three, so that the surprisal curve is a quadratic polynomial.

Although surprisal smoothing requires numerical optimization, convergence is very fast and total surprisal calculation is accomplished in seconds since surprisal surfaces are only mildly non-planar. After surprisal curves are estimated the total arc length of the test information curve is computed.

## Empirical Illustration: The Swedish SAT

**Figure 5.**
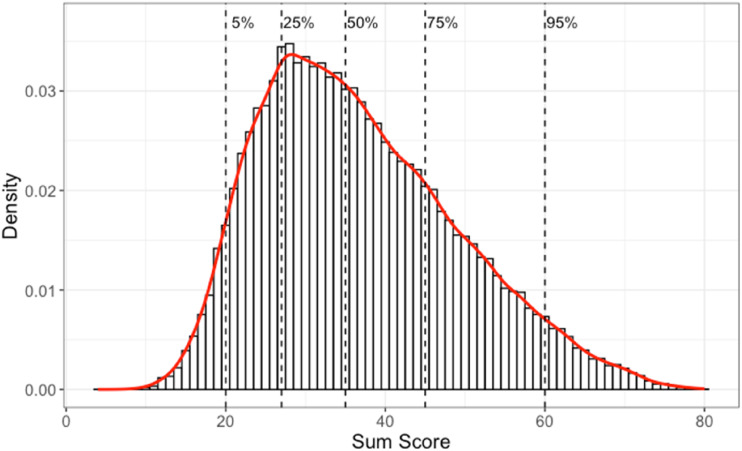
The sum score distribution for the SweSAT-Q 13B test, with a histogram and a red overlaid smooth line. The vertical dashed lines indicate five quantiles (5%, 25%, 50%, 75%, and 95%) as indicated.

Data used in this study is of the quantitative part of the college admissions test, Swedish Scholastic Aptitude Test (SweSAT), in 2013, with 53,768 test takers, and is referred as the SweSAT-Q 13B. In this test, the 80 multiple-choice items had four choices for 68 items and five for 12 items. We added an extra option to each of the 80 items to represent invalid or missing responses and therefore worked with a total of 412 options. These items were designed to represent four types of quantitative expertise: mathematical problem solving (24 items), quantitative comparisons (20 items), quantitative reasoning (12 items) and extracting information from diagrams, tables, and maps (24 items). Figure 5 displays this test’s distribution of sum scores, indicating that this was a difficult test: the median score was 35 out of 80, only two test takers obtained perfect scores; and test takers at the 95% percentile failed 1/4 of the items.

The results in this section will be for the SweSAT-Q 13B test analyzed using both the nominal model using the R package mirt (Chalmers, 2012) and the proposed methodology using TestGardener (Ramsay & Li, 2021), where the later package also has a web application described in Li et al. (2019). The two objects required from mirt are a matrix containing parameter estimates and a vector containing estimated values of θ, which are closely bounded within the interval [−2.5, 4.0].

**Figure 6.**
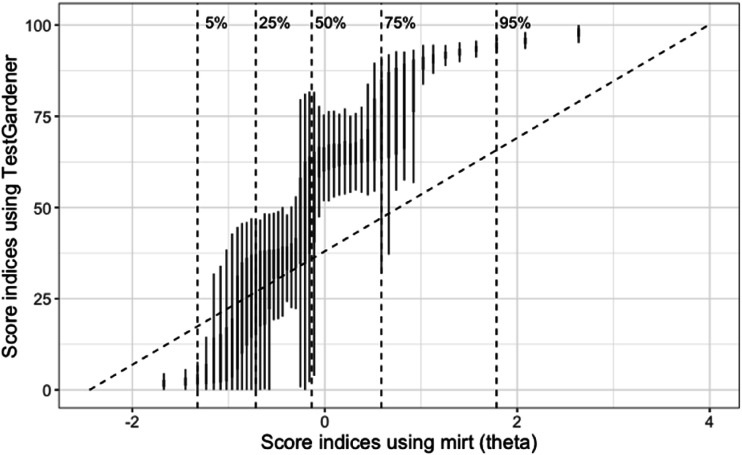
Relationship between the score indices (*θ*) estimated using the nominal model (mirt, x-axis) and TestGardener model (y-axis). Instead of using scatter plot of the 53,768 examinees, we binned the nominal *θ* values using 50 bins, and using boxplot to show the distribution of TestGardener *θ* values in each bin. The vertical dashed lines indicate five quantiles (5%, 25%, 50%, 75%, and 95%) as indicated.

Figure 6 shows the relationship between individual *θ* estimates using the two methods. It can be concluded that the two *θ*s generally correlated with some noteworthy observations: the TestGardener estimated score indices had much larger variation in the middle than at both ends, where examinees were more similar that they got most items wrong (lower end) or right (upper end). The nominal *θ*s were right skewed as sum scores, where the top 25% examinees had a large difference in their nominal *θ*s. However, the TestGardener method assigned the top examinees with much closer score indices, and by doing that, it reduces the influence of ill-performing items, as will be discussed later.

Twenty cycles were used, over which the mean of $F\left({\hat{\theta }}_{j}\right)$ decreased smoothly from 57.0 for the percentage rank initial values to 56.3 for the converged result, and the arc length of the test information manifold increased from the initial value of 34.8 *M*-bits, defined by sum scores percentage ranks, to 78.2 *M*-bits. The nominal arc length was 25.5 *M*-bits. Since arc length is on an additive scale and indicates the amount of information covered by the test questions (see *Scope: Arc lengths* $d_{i}\left(\theta \right)$ and $d_{I}\left(\theta \right)$ *in the curve manifold* $M_{I}$ ), we can conclude that our representation of the data contains over twice the content as those of either the sum scores or the nominal model scores.

**Figure 7.**
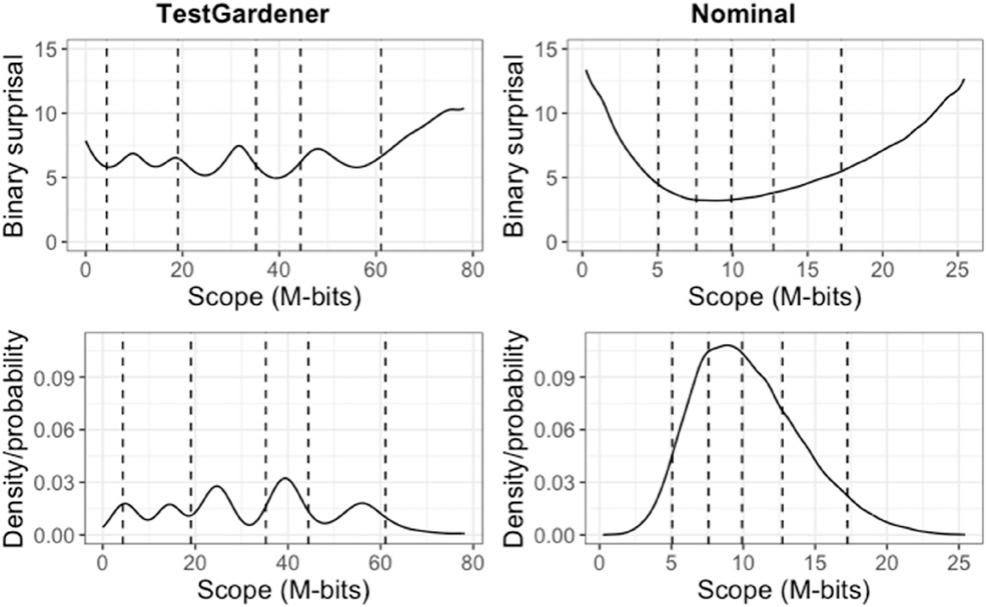
The density of *θ* displayed in terms of surprisal and probability for the two models. The bottom row shows the score density (as proportion/possibility) of score indices (*θ*) of the corresponding model. The top row transforms the corresponding density/probability into binary surprisal values. The vertical dashed lines indicate five quantiles (5%, 25%, 50%, 75%, and 95%) as indicated.

**Figure 8.**
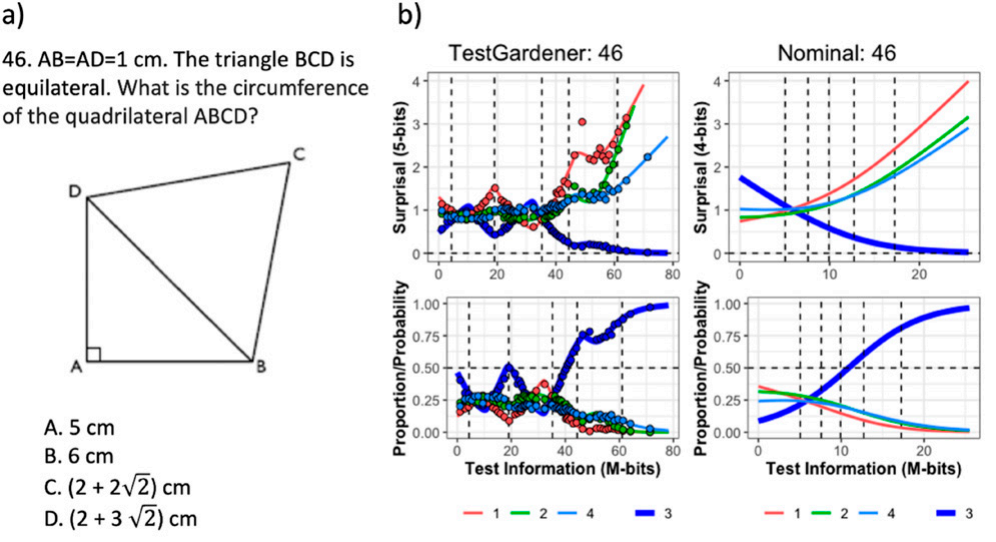
The question (a) and ICCs (b) of item 46. The top row in panel b displays the surprisal curves for the TestGardener and the nominal analyses, respectively. The bottom panels show the corresponding probability curves. The correct answer in each panel is the thick blue line, and the three incorrect answers are shown as thin lines. The curves for the missing or illegitimate responses are omitted. The bin centers are shown as points for the TestGardener analysis. The abscissa is the total test arc length measure for the respective models. The vertical dashed lines indicate five quantiles (5%, 25%, 50%, 75%, and 95%) as indicated.

**Figure 9.**
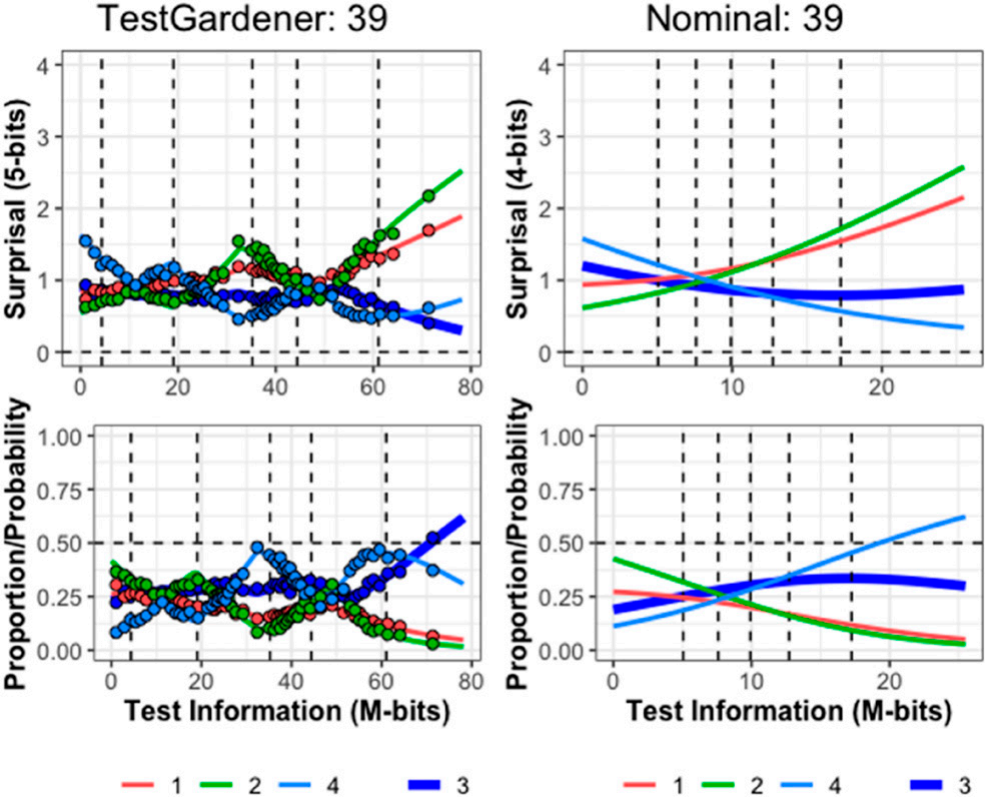
ICCs of item 39. Item 39 required calculating percentage change for a tabled time series. We cannot show the exact item due to copyright. The top row displays the surprisal curves for the TestGardener and the nominal analyses, respectively. The bottom panels show the corresponding probability curves. The correct answer in each panel is the thick blue line, and the three incorrect answers are shown as thin lines. The curves for the missing or illegitimate responses are omitted. The bin centers are shown as points for the TestGardener analysis. The abscissa is the total test arc length measure for the respective models. The vertical dashed lines indicate five quantiles (5%, 25%, 50%, 75%, and 95%) as indicated.

**Figure 10.**
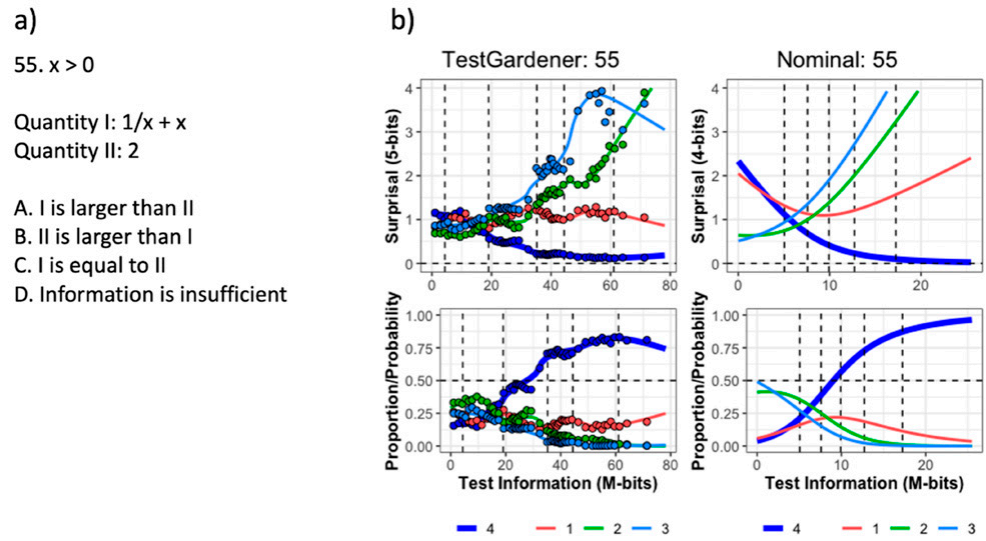
The question (a) and ICCs (b) of item 55. The top row in panel b displays the surprisal curves for the TestGardener and the nominal analyses, respectively. The bottom panels show the corresponding probability curves. The correct answer in each panel is the thick blue line, and the three incorrect answers are shown as thin lines. The curves for the missing or illegitimate responses are omitted. The bin centers are shown as points for the TestGardener analysis. The abscissa is the total test arc length measure for the respective models. The vertical dashed lines indicate five quantiles (5%, 25%, 50%, 75%, and 95%) as indicated.

In the four-panel displays that follow (Figures 7–10), the abscissa is the test information interval, which is [0,78.2] for our model and [0,25.5] for the nominal model. This choice was made because test information is invariant over smooth monotone transformations of the score index and is an additive scale. The upper two panels use surprisal as the ordinate for the same reasons, and the lower two panels use probability. The left panels display results for the proposed TestGardener model and the right for the nominal model.

### Test Information Densities

The test information score densities are represented in Figure 7 as a solid line. Both the surprisal and probability variation for our model indicate the existence of five clusters, but the probability variation is greatly exaggerated relative to that for the linear surprisal scale, where the variation is relatively limited around six 2-bits for all but the highest 5% of test takers. The nominal model displays for both quantities a simpler variation that is more concentrated in the central region.

Our method assigned 81 and 123 test takers to scores 0 and 78.2, respectively. That is, the effects of guessing for the weaker test takers and the handicaps of the two flawed items for the stronger test takers have been effectively removed. The nominal model was not able to accommodate nonzero boundary scores because of the structure of the model. Both methods placed the 5% marker much closer to the left boundary than the 95% marker is to the right boundary, indicating that the stronger test takers are absorbing information much faster than their weaker counterparts.

### The Option Curves for Three SweSAT-Q Items

Figure 8 displays the converged surprisal and probability curves for the relatively difficult item number 46 (panel a). In panel b, the left two plots indicate that the correct answer, C, with the thick blue curve, does not proceed monotonically to 0 for surprisal and 1 for probability. There are two reasons for this. Test takers are counselled to choose item C if they have no idea what to choose, and for this item those at around 20% are rewarded for doing so. At about 55 4-bits the correct choice loses some of its support to the green D curve as these test takers realize that the circumference has something to do with $\sqrt{2}$ , but are not sure if “circumference” includes the inner hypotenuse line. These local non-monotonicities are a frequent feature in the TestGardener plots, but are not possible in the nominal model because of its inflexibility. For all curves, we see that the binned data are closely tracked by the seven basis functions.

In this and the next two figures, the one-parameter-per-curve nominal model provides only crude summaries of the surprisal and probability curves compared with the TestGardener panels. The nominal surprisal curves display only a single direction of curvature, and as such define item arc length values of little interpretive value. The surprisal lengths for the more complex item curves vary over the 80 items from 5.0 to 38.8 *M*-bits, which are the primary scalar summary of item power or quality for defining test taker positions within the test information manifold.

Figure 9 indicates that item 39 has two preferred answers for even the strongest test takers, and their curves terminate on the right near surprisal 0.5 *M*-bits and probability 0.5. The item required the computation of a percent increase in a time series. The answer scored right used the previous value as the baseline for an increase, but a substantial portion of even the top performers used baseline zero. It is conjectured that not all secondary school teachers got around to covering the baseline issue.

Item 55 in Figure 10 is an interesting case (panel a). Answer D was designated as right by the test designers, but a bright test taker should have no trouble seeing that the function is greater than two everywhere except at *x* = 1. It seems that a sizeable number of the best test takers saw answer A as the best of confusing choices. Note that the nominal scale model is unable to reveal the real failure rate for the best test takers because its correct answer curve must progress monotonically on the right because of the model’s use of the exponential function. This strong tendency to monotonicity is the price paid for the nominal model’s simplicity, as compared to the functional fits.

The *θ*-derivatives of a test taker’s surprisal curves in the data fit gradient (9) provide direct push-pull pressures on *θ* in order to identify the value that best represents his/her choices. Consequently, we can explore which items are most important in this regard by examining lengths of the item information curves for each item. The four longest trajectories for the 13B test over the high end θ-interval [80,100] were 43, 41, 46, and 61, with arc lengths 31.2. 28.5, 26.6, and 20.1 *M*-bits, respectively.

### Exploring Mutual Information

By summing over item information in equations (7) and (9), we implicitly ignore that the choices for two items *i* and ℓ may be dependent in some sense and therefore share information or, in the language of information theory, possess mutual information. We are here quantifying mutual information between two probability or surprisal matrices, **S**_*i*_(*θ*) and **S**_ℓ_(*θ*) across some replication factor such as test takers.

Table 1 shows in the off-diagonals the values of the mutual entropy *I*_*i*ℓ_ for the pairs of the four most important items for the top 20% of the test takers. In the diagonals are the self-entropy *H*_*i*_ of the items. We see here that the mutual entropy for the pairs is a tiny fraction of the item self-entropy. Highly similar results were found using all 80 items, and we can conclude that defining the length of the test information curve using addition across items is a reasonable procedure.

**Table 1.** The SweSAT-Q 13B Mutual Entropy I_i,ℓ_ for the Four Most Informative Items and the Top 20% of the Test Takers. Diagonal Values are Simple Entropies *H*_*i*_ for Single Items, Which are Also the Values of Mutual Entropies for Two Identical Items.

Item	41	43	46	61
41	0.754	0.001	0.002	0.001
43	0.001	0.235	0.002	0.002
46	0.002	0.002	0.685	0.002
61	0.001	0.002	0.002	0.621

### Plotting the Test Information Manifold

**Figure 11.**
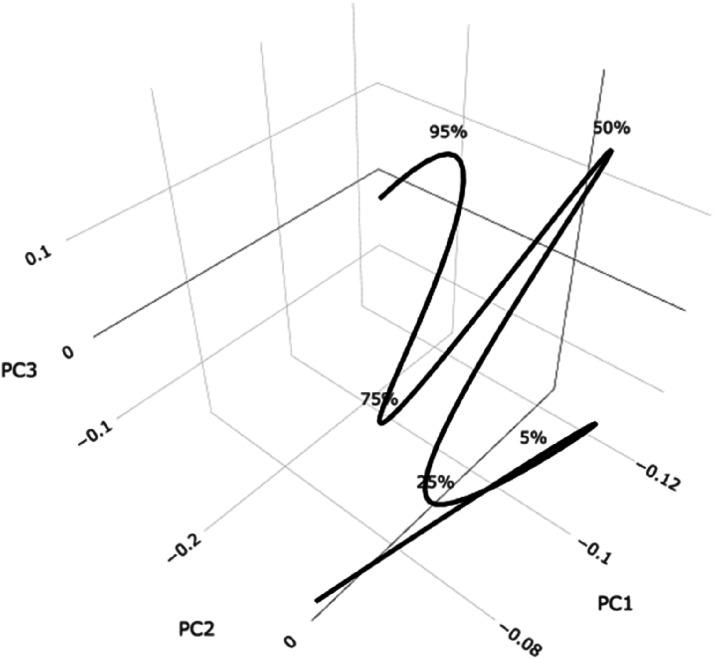
Almost 100% of the shape variation in the test information curve is shown in the three-dimensional plot. The five marker percentages indicate the proportions of test takers at or below their positions. The curve is almost two-dimensional or planar above the 5% point.

Although the test information manifold is embedded in a space of 412 dimensions, almost 100% of the shape variation in the test information curve can be viewed by using the first three singular value functions of the space curve. Figure 11 displays this $M_{S}$ curve, for the 13B test. Above the first 5% of test takers the curve is very nearly two-dimensional. The sharp peak at 50% is probably due to the widely held belief that option C is the most probably correct if one must make a random choice. The six curve portions coincide with the peaks in the density functions in Figure 7.

## Discussion and Conclusions

Our fundamental goal is revealing the structure in test or scale data in a manner that permits easy understanding and assessment through the use of additive scales that are natural for persons possessing all levels of mathematical skill. To achieve this objective, it is essential to have a model system that is easily adjustable to what the data require. Our model is every bit as parametric as the nominal and all of its cousins are, and the displays for the large SweSAT data set indicate clearly that there is more useful and interesting structure than can be accommodated by one parameter per option. The quality of an item is nicely captured by its arc length within information space, and the knowledge level of a test taker is defined in the same way. In this way, two test administrations can be directly compared, as can two tests with differing lengths.

What we have achieved, is a quantitative lens through which we can examine variation in knowledge acquisition. Even elementary models can reveal important information and play a positive role in advancing science. The nominal model for these data may not be entirely adequate, but it also captures important item performance features, and its estimates of test taker performance are useful for initializing a more powerful analysis. What is essential is that data structure be communicated to an observer in a manner that is consistent with the visual system’s linear scanning, and probability is not always appropriate for this.

Equation (7) reveals that data structures are reflected by their way of concatenating surprisal slopes. Information has a simple relation to change; the more the change across *θ* or any other score index, the greater the information. In this way, information is very much like standard deviation as a measure. What information actually means to an observer is determined by how the initial architectures of manifolds $M_{i}$ and $M_{I}$ are defined. We initialized our analyses by a simple transform of the sum score because that score has face validity, and therefore the credentials necessary to allow test takers and test assessors to assume that they are looking at quantitative knowledge, even if initially at the primitive level of binary data. We recommend beginning with sum scores, then moving to a credible binary parametric IRT model such as the two-parameter logistic, followed by a version of the nominal full-data model before using our arbitrarily powerful methodology.

We also need to be more careful about whether a single information number is an appropriate and adequate summary of test taker performance. We see many cases where being able to view the value of the entire function *F*, whether over *θ* or over arc length, is preferred. These cases may be, for example, ones where some aspects of a topic are easily grasped but others not, perhaps even because of failings in their educational processes. Item 39 in the SweSAT is an illustration of this.

Also, switching from probability to surprisal brings a computing benefit that makes analyses of large amounts test and scale data practical on even modest computers. Using surprisal to define the arc length along the test information space curve offers a metric tool, the *M*-bit. The value of fitting criterion function *F* is determined by a simple inner product, and its gradient by a sum weighted by surprisal slope, although it must be remembered that surprisal slope changes from one iteration to another.

The use of arc length also brings other benefits of using *θ*. Lord (1975) discusses how Fisher information, which is commonly utilized within IRT to explore for which values of *θ* a test or item measures well, changes when the arbitrary *θ*-scale is rescaled. This is a fundamental problem that occurs since Fisher information is based on the derivatives with respect to *θ*. By using arc length as a measure of ability, the latent scale is grounded in the test itself and thus one does not have this issue.

A topic for future research, which we have just started exploring, is two-dimensional *θ* structures defined by finite elements over a triangular mesh, using methods reported in Ramsay (2017). The resolution of the mesh can be varied in order to control the number of parameters defining the manifold $M_{S}$ , which is now a surface rather than a curve. This score index structure is designed to recognize that the dimension of *θ* must collapse to 0 for test takers below or above the test. Subsequent versions can be constructed using tessellations of even higher dimensional score index sets.

We do not intend to eliminate the use of probability, which is readily available through the inverse of the surprisal transform, as an informative tool. The familiar probability plots of option, item and test performance remain as useful as always, and especially to ensure that the right answer is doing its job. But we do offer a way to quantify knowledge, performance, and via rating scales subjective experience.

Finally, supplementary materials can be found at https://github.com/JuanLiOHRI/SweSAT13_B, where codes and all 80 four-panel figures for both models, and also a randomly selected set of 100 plots of *F*(*θ*) are given.

## Supplemental Material

Supplemental Material - An Information Manifold Perspective for Analyzing Test Data Supplemental Material for An Information Manifold Perspective for Analyzing Test Data by James Ramsay, Juan Li, Joakim Wallmark, and Marie Wiberg in Applied Psychological Measurement.

## References

[bibr1-01466216241310600] Bock R. D. (1972). Estimating item parameters and latent ability when responses are scored in two or more latent categories. Psychometrika, 37(1), 29–51.

[bibr2-01466216241310600] Briggs D. C. Alonzo A. C. Schwab C. Wilson M. (2006). Diagnostic assessment with ordered multiple-choice items. Educational Assessment, 11(1), 33–63. 10.1207/s15326977ea1101_2

[bibr3-01466216241310600] Chalmers R. P. (2012). Mirt: A multidimensional item response theory package for the R environment. Journal of Statistical Software, 48(6), 1–29. 10.18637/jss.v048.i06

[bibr4-01466216241310600] Cover T. M. Thomas J. A. (2006). Elements of information theory. Wiley-Interscience.

[bibr5-01466216241310600] Fisher R. A. (1922). On the mathematical foundations of theoretical statistics. Philosophical Transactions of the Royal Society of London,Series A, 222(1), 309–368.

[bibr31-01466216241310600] Haberman S. J. Liu Y. Lee Y-H. (2019). Distractor analysis for multiple-choice tests: an emprical study with international language assessment data. ETS Research report, 19(39).

[bibr6-01466216241310600] Kullback S. (1959). Information theory and statistics: Wiley.

[bibr7-01466216241310600] Li J. Ramsay J. O. Wiberg M. (2019). TestGardener: A program for optimal scoring and graphical analysis. In Wiberg M. Culpepper S. Janssen R. González J. Molenaar D. (Eds.), Quantitative psychology. IMPS 2017. Springer proceedings in mathematics and statistics (265, pp. 87–94): Springer. 10.1007/978-3-030-01310-3_8

[bibr8-01466216241310600] Lord F. M. (1975). The ‘ability’ scale in item characteristic curve theory. Psychometrika, 40(2), 205–217. 10.1007/bf02291567

[bibr9-01466216241310600] Luce R. D. (1959). Individual choice behavior: A theoretical analysis: Wiley.

[bibr12-01466216241310600] Ramsay J. O. (1991). Kernel smoothing approaches to nonparametric item characteristic curve estimation. Psychometrika, 56(4), 611–630. 10.1007/bf02294494

[bibr13-01466216241310600] Ramsay J. O. (2017). A functional estimate of covariation. Journal of Computational & Graphical Statistics, 26(1), 160–170. 10.1080/10618600.2015.1124041

[bibr15-01466216241310600] Ramsay J. O. Li J. (2021). TestGardener: Optimal analysis of test and rating scale data. R package version 2.0.1. https://CRAN.Rproject.org/package=TestGardener

[bibr16-01466216241310600] Ramsay J. O. Li J. Wiberg M. (2020b). Better rating scale scores with information-based psychometrics. The Psychologist, 2(4), 347–369. 10.3390/psych2040026

[bibr19-01466216241310600] Ramsay J. O. Wiberg M. Li J. (2020a). Full information optimal scoring. Journal of Educational and Behavioral Statistics, 45(3), 297–315. 10.3102/1076998619885636

[bibr20-01466216241310600] Rossi N. Wang X. Ramsay J. O. (2002). Nonparametric item response function estimates with the EM algorithm. Journal of Educational and Behavioral Statistics, 27(3), 291–317. 10.3102/10769986027003291

[bibr21-01466216241310600] Shannon C. (1948). A mathematical theory of communication. Bell System Technical Journal, 27(3), 379–423. 10.1002/j.1538-7305.1948.tb01338.x

[bibr22-01466216241310600] Stone J. V. (2022). Information theory: A tutorial introduction (2nd ed.). Sebtel Press.

[bibr23-01466216241310600] Suh Y. Bolt D. M. (2010). Nested logit models for multiple-choice item response data. Psychometrika, 75(3), 454–473. 10.1007/s11336-010-9163-7

[bibr24-01466216241310600] Thissen D. Cai L. (2016). Nominal categories models. In van der Linden W. (Ed.), Handbook of item response theory, volume 1, models. CRC Press.

[bibr25-01466216241310600] Thissen D. Steinberg L. (1984). A response model for multiple choice items. Psychometrika, 49(4), 501–519. 10.1007/bf02302588

[bibr26-01466216241310600] Thissen D. Steinberg L. Fitzpatrick A. R. (1989). Multiple-choice models: The distractors are also part of the item. Journal of Educational Measurement, 26(2), 161–176. 10.1111/j.1745-3984.1989.tb00326.x

[bibr27-01466216241310600] Tribus M. (1961). Thermodynamics and Thermostatistics: An introduction to energy, information and states of matter, with engineering applications: D. Van Nostrand.

[bibr28-01466216241310600] van der Linden W. (Ed.), (2016). Handbook of item response theory, volume 1, models. CRC Press.

[bibr29-01466216241310600] Wiberg M. Ramsay J. O. Li J. (2019). Optimal scores – an alternative to parametric item response theory and sum scores. Psychometrika, 84(1), 310–322. 10.1007/s11336-018-9639-4 30350132

[bibr30-01466216241310600] Wilson M. (1992). The ordered artition model: An extension of the partial credit model. Applied Psychological Measurement, 16(4), 309–325. 10.1177/014662169201600401

