# Supplementary material for: An Information Manifold Perspective for Analyzing Test Data
Source: Appl Psychol Meas. 2024 Dec 20;49(3):90–108. doi: 10.1177/01466216241310600 (PMC11662344; doi:10.1177/01466216241310600)
Supplement: Supplemental Material - An Information Manifold Perspective for Analyzing Test Data [file sj-pdf-1-apm-10.1177_01466216241310600.pdf]

APM: Final Manuscript Submission Checklist

Please note that all materials should, if possible, be submitted online, as a revision of your manuscript. This allows the manuscript's final files to proceed directly, via the Manuscript Central System, to Production after the Editor has completed his final check. It is not necessary any longer to send electronic files to us via e-mail.

(X) Author Contact Information for all authors if possible, and for the corresponding author at the absolute minimum, containing up-to-date contact information for each author, including:

- Institutional affiliation, with desired affiliation to be printed with the manuscript if different from current affiliation
- Work address
- Work phone number
- Work fax number
- Work e-mail address
- Home address*
- Home phone number*
- Other e-mail address (if any)*

(X) The final version of the manuscript (preferably in MS Word format; if submitting in LaTex/Tex format, please follow the LaTex/Tex submission instructions attached separately).

(X) Electronic files for all figures (camera-ready; see attachment for information on preparing camera-ready figures: "SAGE Artwork Submission Guidelines.")

(X) Abstract (should be part of the manuscript itself)

(X) Keywords (should be part of the manuscript itself)

(X) Tables (should be part of the manuscript itself). Also please make sure all the tables and equations are in their raw formats (word, excel, LaTex, etc.), not as images. We cannot accept tables or equations in image format because we are unable to edit them and they can become disfigured.

(X) References (should be part of the manuscript itself)

(X) Acknowledgements, if any (should be part of the manuscript itself)

(NA) Appendices, if any (If the appendices are accepted as a part of the main article, they must be submitted as a part of the Main Document; if they are accepted as online supplements, they must be submitted as a separate Supplementary File.)

(NA) Permission to use any reproduced or copyrighted material (if needed)

(X) This checklist, completed.

* Needed to facilitate communications in case of a change in institutional affiliation. Will not be used unless contact cannot be made using work information. (Since Sage Publishers is working during vacations, weekends, etc., they like to have alternative contact information available in case something urgent arises and you are not at work.)
